# Nonbacterial Thrombotic Endocarditis: Presentation, Pathophysiology, Diagnosis and Management

**DOI:** 10.31083/j.rcm2304137

**Published:** 2022-04-11

**Authors:** Shahad Al Chalaby, Rakhee R Makhija, Ajay N. Sharma, Muhammad Majid, Edris Aman, Sandhya Venugopal, Ezra A. Amsterdam

**Affiliations:** ^1^Alameda Health System – Highland Hospital, Oakland, CA 94602, USA; ^2^Division of Cardiovascular Medicine, University of California Davis, Davis, CA 95817, USA; ^3^School of Medicine, University of California Irvine, Irvine, CA 92697, USA

**Keywords:** nonbacterial thrombotic endocarditis, marantic endocarditis, Libman-Sacks endocarditis, endocarditis

## Abstract

Initially described in 1936, non-bacterial thrombotic endocarditis (NBTE) is a rare entity involving sterile vegetations on cardiac valves. These vegetations are usually small and friable, typically associated with hypercoagulable states of malignancy and inflammatory diseases such as systemic lupus erythematosus. Diagnosis remains challenging and is commonly made post-mortem although standard clinical methods such as echocardiography (transthoracic and transesophageal) and magnetic resonance imaging may yield the clinical diagnosis. Prognosis of NBTE is poor with very high morbidity and mortality usually related to the serious underlying conditions and high rates of systemic embolization. Therapeutic anticoagulation with unfractionated heparin has been described as useful for short term prevention of recurrent embolic events in patients with NBTE but there are no guidelines for management of this disease.

## 1. Introduction

Nonbacterial thrombotic endocarditis (NBTE) was initially described by Gross and Friedberg in 1936 in an extension of the original classification proposed by Libman in 1923 [1, 2]. Subsequently, NBTE has also been termed verrucous endocarditis or marantic endocarditis. “Marantic” from Greek, *marantikos*, means “wasting away”, due to association of NBTE with grave diseases such as cancer. The term, Libman-Sacks endocarditis, specifically refers to NBTE associated with systemic lupus erythematosus (SLE). Thus far, the etiology of NBTE is uncertain and postulated to be secondary to inflammatory and hypercoagulable states [3, 4, 5].

## 2. Etiology

NBTE is most frequently associated with neoplasia (32–80%) in postmortem studies and its association with SLE has been reported as approximately 10% based on echocardiographic criteria [3, 4, 5, 6]. Other etiologies of NBTE include antiphospholipid syndrome (APS), rheumatoid arthritis, Behcet’s disease, adult-onset Still’s disease, systemic hypereosinophilic syndrome, and severe burn cases [3, 7, 8, 9, 10, 11, 12]. NBTE has also been reported with other conditions including Waldenstrom macroglobulinemia [13], multiple myeloma [14], hyperhomocysteinemia [15], and Crohn’s disease [16]. Additionally, recent cases have been related to clinical and subclinical COVID19 infection [17, 18].

## 3. Epidemiology 

NBTE is found in approximately 1.0% of all autopsies, most commonly occurring from the fourth through eighth decades of life and equally affecting males and females [7, 19]. Prevalence varies widely in clinical reports (0.3% to 9.0%), likely due to differences in underlying conditions and variability of diagnostic methods [7, 19]. In an autopsy series, NBTE was more common in patients with, than without, cancer (~1.0% vs. ~0.2%; *p *< 0.05) [8]. Its occurrence is higher in patients with adenocarcinoma (lung, ovary, biliary, prostate) than with other malignancies (2.7% vs. 0.5%; *p *< 0.05), especially in cases of pancreatic cancer (10%) [8].

Systemic embolization is the most common clinical manifestation of NBTE [5, 8, 9, 20]. In a postmortem series of patients with cancer and stroke, the prevalence of NBTE was 32% [21]. Similarly, in a prospective transthoracic echocardiographic (TTE) series of 200 patients with solid tumors, the frequency of NBTE was 19% compared to only 2.0% in cases without cancer who were referred for TTE to detect the source of an occult arterial thromboembolic event (*p *< 0.001) [22]. Further, in patients without overt cardiac disease who sustained an occult arterial embolism, evidence of the latter event was present in 24% of those with solid tumors compared to 8% in control patients (*p* = 0.01) [22]. With respect to strokes, a retrospective evaluation of 51 cancer patients with cerebral ischemic infarcts revealed NBTE in 18% based on transesophageal echocardiography (TEE) [23]. Of those with cardiac vegetations, 47% definitively experienced a cerebrovascular event, often with disabling anterior circulation strokes or infarcts in multiple vascular territories [23]. Cardiac valvular specimens obtained at surgery from 30 patients with NBTE revealed immune-mediated disorders in 60% of this group [24]. Underlying conditions included primary APS, rheumatic heart disease, SLE, and rheumatoid arthritis. Importantly, no patient had evidence of malignancy or intravascular coagulation in this surgical population.

## 4. Pathogenesis

NBTE vegetations primarily consist of agglutinated blood and platelet thrombi interwoven with strands of fibrin, immune complexes, and mononuclear cells (“white thrombus”) [24]. The friability and embolization of these lesions appear to be related to lack of inflammatory adhesions, with the frequency of visceral embolism varying very widely (14–91%) [25]. These lesions, which differ considerably in size, are classically found in areas of high flow on valve leaflets and are usually situated along valve closure lines. A classification has been proposed based on size and verrucal characteristics [26]: type 1—small (<3 mm diameter), univerrucal, firmly attached to valve; type 2—large (>3 mm), univerrucal, adherent to valve; type 3—small (1–3 mm), multiverrucal, friable; type 4—large (>3 mm), multiverrucal, friable; type 5—‘healed’, similar in consistency to attached valve. In multiple autopsy series (totaling 119 patients), 75% of the valvular lesions were <3 mm in diameter and 70% were multiverrucal [25, 27, 28, 29].

The inciting event for development of NBTE is not well understood, although it appears related to endothelial damage in the setting of hypercoagulable and inflammatory states occurring in a variety of autoimmune, infectious, inflammatory and malignant conditions. Additionally, some observational and experimental studies have shown that hypoxia is associated with increased risk of thrombosis [30] which might explain recent evidence of NBTE in patients with COVID19 infection [17, 18]. In an experimental study, clot provoking tissue factor (TF) was induced in cardiac valvular cells by exposure to hypoxic conditions which resulted in NBTE-like valvular lesions [31]. In a recent study of 100 patients with COVID19 infection and 28 healthy controls, TF activity levels were significantly higher in patients with COVID19 compared with controls (*p *< 0.0001). Higher TF levels correlated with thrombosis and were linked to greater COVID19 disease severity and mortality [32]. Two recent reviews hypothesized that induction of TF expression may play a significant role in COVID19 related thrombosis [33, 34]. Whether high levels of TF in patients with COVID19 infection increases risk of NBTE has not been determined.

## 5. Presentation 

Symptoms identified at the time of NBTE diagnosis such as fever, are usually nonspecific and related to underlying conditions. NBTE itself produces no symptoms until the occurrence of emboli. Physical examination may rarely reveal a cardiac murmur caused by valvular dysfunction related to a large vegetation of fibrin-platelet thrombi [24]. The most frequent presentation occurs when the vegetations dislodge and embolize, which ensues in approximately 50% of patients [9, 10]. Embolization is more common than valvular dysfunction because the lesions do not usually produce sufficient valvular impairment to alter valve function [9, 10]. Emboli may present as a cerebrovascular event, myocardial ischemia or infarction, a cold limb, severe abdominal pain due to mesenteric ischemia, or flank pain due to renal arterial emboli.

## 6. Laboratory

The diagnosis of NBTE is suggested by absence of typical and atypical organisms of culture-positive and culture-negative endocarditis, and presence of predisposing conditions such as malignancy, SLE or other inflammatory processes. Diagnosis is often challenging as blood cultures are negative in up to 5% of patients with infective endocarditis (IE) [35] owing to either prior antibiotic therapy or infection with fastidious organisms, such as *Coxiella burnetii*, *Legionella pneumophila*, *Chlamydia trachomatis*, and fungi (candida, histoplasma and aspergillus species). Serologic evaluation for hypercoagulable states, including lupus anticoagulant and disseminated intravascular coagulation panels, should be performed in all patients suspected of having NBTE. Other immunological assays including antiphospholipid antibodies, anticardiolipin antibodies, anti-b2-glycoprotein 1 antibodies (at least one must be positive for the diagnosis of APS on at least two occasions, 12 weeks apart) should be employed in patients presenting with recurrent systemic emboli or known SLE. In addition, age-appropriate screening for cancer should be conducted.

## 7. Imaging

**Fig. 1.**
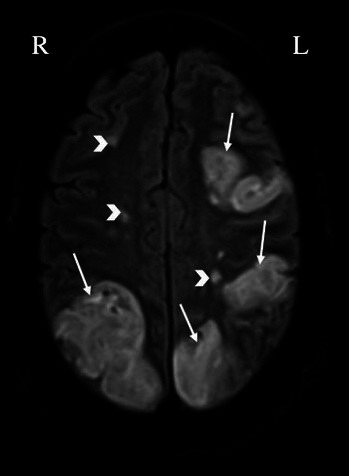
**Evidence of multiple bilateral cerebral infarcts in a patient with nonbacterial thrombotic endocarditis (arrows: large infarcts, arrowheads: smaller infarcts) detected by brain magnetic resonance imaging**. L, left; R, right.

Evidence from computed tomography or magnetic resonance imaging (MRI) of the brain that are consistent with multiple embolic strokes raise consideration of NBTE, as detected in a recent patient of ours with NBTE associated with lung cancer (Fig. 1) [36]. Given the lack of specificity of clinical presentation alone, brain imaging is often necessary in equivocal cases [37, 38]. Diffusion-weighted MRI can help differentiate between cardioembolic strokes resulting from IE and NBTE depending on the stroke pattern [37]. NBTE patients uniformly had multiple, widely distributed strokes of variable sizes (<10 mm to >30 mm). Patients with IE, on the other hand, might have a similar stroke pattern to NBTE or manifest as a single or a territorial cerebral infarction. NBTE may present this pattern of involvement because vegetations associated with this process have little cellular organization and thus a higher potential for fragmentation and widespread embolization [23].

**Fig. 2.**
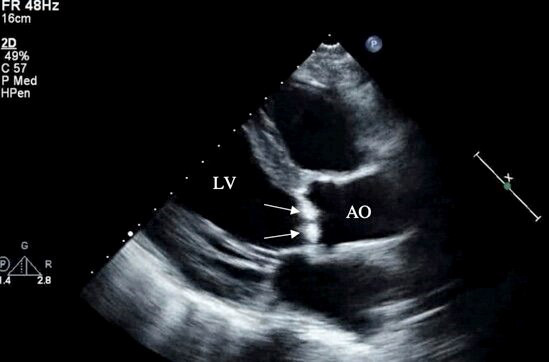
**Transthoracic echocardiography (parasternal long axis view) of a patient with nonbacterial thrombotic endocarditis showing large irregular hyperechoic lesions consistent with vegetations on the aortic valve (arrows)**. LV, left ventricle; AO, ascending aorta.

NBTE vegetations are most frequently left sided, with two-thirds involving the mitral valve and the remainder occurring on the aortic valve. Rarely, both valves can be affected [5, 8, 9, 38]. Due to the small size of the many vegetations seen in NBTE, pathology may frequently escape detection by TTE. In patients in whom there is a high suspicion of NBTE in the setting of an unclear TTE scan, a TEE is indicated [23] (Fig. 2). It has been reported that cardiac MRI may be useful to evaluate valvular vegetations in NBTE [39].

## 8. Diagnosis

**Fig. 3.**
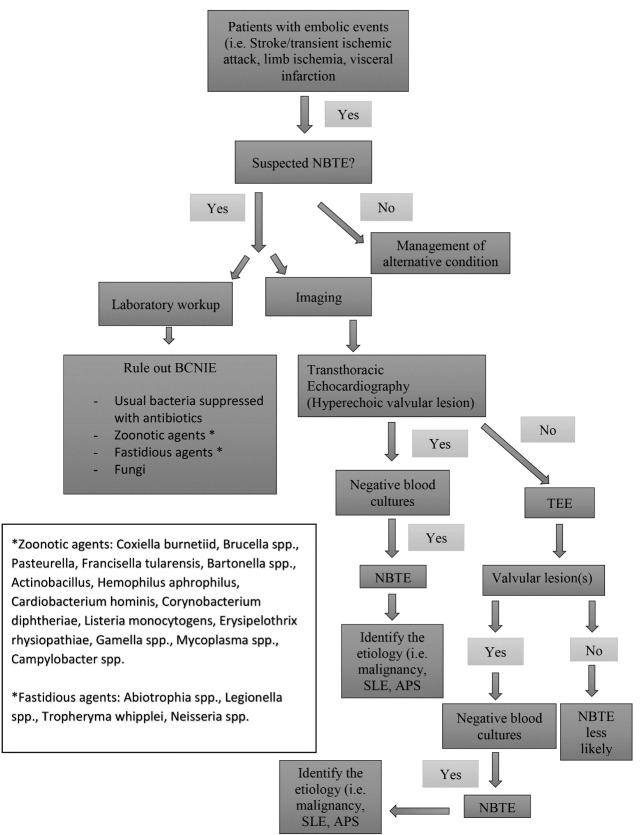
** Algorithm summarizing diagnostic approach to non-infective endocarditis including laboratory tests and imaging**. The algorithm is initiated in response to suggestive clinical findings and proceeds according to the pathways shown, utilizing appropriate laboratory and imaging methods in a stepwise approach to exclude or rule-in potential diagnoses.

Diagnosis requires a high index of suspicion, as risk factors such as malignancy, SLE, or APS may be absent. Blood culture negative infective endocarditis (BCNIE) should be excluded before considering NBTE diagnosis (Fig. 3). Various diagnostic tests including different culture media, serological tests and polymerase chain reaction should be considered to exclude BCNIE [40]. A thorough history is essential in guiding further investigation and identification of the offending microorganism in BCNIE. A history of antibiotic therapy, even as brief as 2 to 3 days prior to blood cultures, should be explored as it is a common cause of BCNIE [40]. A variety of animal exposures, contact with sheep and cattle, human body louse, and cats suggests infection with *Coxiella burnetii*, *Bartonella quintana* and *Bartonella henselae*. Travel to the Middle East and ingestion of unpasteurized milk suggest infection with Brucella species. Legionella should be suspected from a history of recent hospitalization, and fungal infection should be excluded in patients with prolonged antibiotic therapy and immunosuppression [40]. After ruling out BCNIE, patients suspected of having NBTE should be evaluated with TTE, and inconclusive findings followed by TEE. However, even TEE may be unrevealing if only small remnants of prior vegetations remain on cardiac valves.

NBTE and IE cannot be distinguished with imaging alone as there are no pathognomonic echocardiographic features of the vegetations, and diagnosis must include clinical context [25]. Definitive diagnosis is achieved through pathological identification of autopsy or surgical specimens. However, the combination of clinical presentation, valvular vegetations on echocardiography, absence of systemic infection, and underlying serious noncardiac disease, most commonly supports the diagnosis of NBTE. Finally, meticulous attention should address exclusion of IE, as the management of embolic phenomena due to the latter differs considerably from that related to NBTE [9].

## 9. Management

Treatment guidelines for NBTE are not well established and management is often challenging. Detection and treatment of underlying disease (e.g., malignancy, SLE, APS) is crucial; however, such therapy is not known to clear the valvular vegetations [4]. Nevertheless, in isolated cases such as those with SLE, it has been suggested that steroids may be beneficial [41, 42]. The anticoagulant of choice to prevent recurrent thrombosis in patients with SLE and APS is warfarin after a period of anticoagulation with LMWH or unfractionated heparin [43].

Anticoagulation is critical to prevent recurrent embolization [9, 19]. IE including BCNIE, should be excluded before initiation of anticoagulation as septic emboli have a high propensity to provoke intracranial bleeding [9]. Additionally, hemorrhagic conversion of an embolic stroke related to NBTE should be excluded by computed tomography prior to anticoagulation. After alternative diagnoses are ruled out, intravenous unfractionated heparin or subcutaneous low molecular weight heparin should be administered [4, 9]. Guidelines from the American College of Chest Physicians in 2012, based on observational studies, recommend the use of unfractionated or LMWH for treatment of NBTE and prevention of thromboembolism. There are no controlled trials directly comparing vit K antagonists with heparin for NBTE treatment and little apparent benefit has been observed with warfarin therapy in NBTE [41].

In a study of 182 patients over 3 decades ago with chronic disseminated coagulopathy and malignancy, the authors detected the following findings at autopsy: single or migratory thrombophlebitis, hemorrhage, arterial emboli, and NBTE [44]. In this population, therapy with unfractionated heparin was more beneficial than warfarin [44]. In an autopsy study of 42 documented cases of cerebral infarction from cancer-associated NBTE, continuous unfractionated heparin was the preferred therapeutic agent [45]. Continuous heparin infusion was not associated with recurrent embolic events compared to intermittent dosing, and hemorrhagic transformation of the cerebral infarct was not noted [45]. One large observational study found that coagulopathy and NBTE accounted for 12% and 3% of all strokes, respectively, in patients with cancer [46].

Thrombotic lesions in NBTE may become secondarily infected by circulating bacteria; therefore, despite lack of evidence, preventive antibiotic therapy before procedures known to produce bacteremia may be considered [40, 47, 48]. Surgical valve replacement is not recommended unless recurrent thromboembolism is present despite continuing anticoagulation [48]. Other indications for valve replacement are similar to those for IE [40].

## 10. Follow-Up 

The optimal intervals for follow-up have not been defined. Patients should be monitored for complications of NBTE, such as persistent embolization despite anticoagulation, bleeding, and thrombocytopenia. Repeat echocardiography at regular intervals should also be considered to assess disease progression [4, 48].

## 11. Prognosis

Data from retrospective studies suggest that prognosis is unfavorable despite anticoagulation, with a high morbidity and mortality primarily due to a strong association with the primary condition, such as advanced cancer. In this regard, in 30 patients with SLE, a six-year cross-sectional study reported poor outcomes due to recurrent stroke (25%), cognitive disability (24%), and death (9%) [4].

## 12. Future Directions 

Incorporating 3D echocardiography in contemporary TEE evaluation of suspected NBTE cases should be considered and further investigated in future studies given the promising findings from a recent retrospective study supporting its utility in NBTE diagnosis [49]. Cardiac MRI has similar sensitivity, specificity and diagnostic accuracy to TEE in the detection of valvular vegetations, but a greater capacity for tissue differentiation and characterization including identification of endocardial inflammation [50]. In addition to its 3D capability, cardiac MRI is a non-invasive imaging modality that has important potential for future implications in the diagnosis of NBTE; hence, future studies directly comparing the use of TEE and cardiac MRI are warranted. To date, heparin (LMWH or unfractionated) is the anticoagulant of choice in NBTE based on limited observational studies [41]. Prospective studies and clinical trials directly comparing heparin with warfarin and direct oral anticoagulants are necessary to guide future directions in NBTE management.

## 13. Conclusions

Non-bacterial thrombotic endocarditis is commonly associated with malignancies, systemic lupus erythematosus, and other severe autoimmune and inflammatory diseases. Morbidity and mortality are usually high due to systemic embolization from friable cardiac valve vegetations and underlying diseases. Despite improvement in diagnostic methods, such as transesophageal echocardiography and cardiac MRI, diagnosis remains challenging, and treatment is limited. Valvular intervention by surgery or catheter approach is rarely indicated. There are no guidelines for management of NBTE. Prognosis of NBTE remains grave due to advanced underlying disease and/or neurologic dysfunction from disabling embolic strokes.
